# Predicting decompression surgery by applying multimodal deep learning to patients’ structured and unstructured health data

**DOI:** 10.1186/s12911-022-02096-x

**Published:** 2023-01-06

**Authors:** Chethan Jujjavarapu, Pradeep Suri, Vikas Pejaver, Janna Friedly, Laura S. Gold, Eric Meier, Trevor Cohen, Sean D. Mooney, Patrick J. Heagerty, Jeffrey G. Jarvik

**Affiliations:** 1 Department of Biomedical Informatics and Medical Education, School of Medicine, University of Washington, Box 358047, Seattle, WA 98195 USA; 2 Clinical Learning, Evidence and Research Center, University of Washington, 4333 Brooklyn Ave NE, Seattle, WA 98105 USA; 3 Department of Rehabilitation Medicine, University of Washington, 1959 NE Pacific St, Seattle, WA 98195 USA; 4 Institute for Genomic Health, Icahn School of Medicine at Mount Sinai, New York, NY 10029 USA; 5 Department of Genetics and Genomic Sciences, Icahn School of Medicine at Mount Sinai, New York, NY 10029 USA; 6 Department of Radiology, University of Washington, 1959 NE Pacific Street, Seattle, WA 98195 USA; 7 Department of Biostatistics, University of Washington, Box 357232, Seattle, WA 98195-7232 USA; 8 Center for Biomedical Statistics, University of Washington, Seattle, WA USA; 9 Department of Neurological Surgery, University of Washington, 1959 NE Pacific Street, Seattle, WA 98195 USA; 10 Department of Health Services, University of Washington, Box 357660, Seattle, WA 98195-7660 USA

**Keywords:** Lower back pain, Lumbar spinal stenosis, Lumbar disc herniation, Deep learning, Generalizability, Multimodal, Machine learning, Decompression surgery, Prediction, Classification

## Abstract

**Background:**

Low back pain (LBP) is a common condition made up of a variety of anatomic and clinical subtypes. Lumbar disc herniation (LDH) and lumbar spinal stenosis (LSS) are two subtypes highly associated with LBP. Patients with LDH/LSS are often started with non-surgical treatments and if those are not effective then go on to have decompression surgery. However, recommendation of surgery is complicated as the outcome may depend on the patient’s health characteristics. We developed a deep learning (DL) model to predict decompression surgery for patients with LDH/LSS.

**Materials and method:**

We used datasets of 8387 and 8620 patients from a prospective study that collected data from four healthcare systems to predict early (within 2 months) and late surgery (within 12 months after a 2 month gap), respectively. We developed a DL model to use patients’ demographics, diagnosis and procedure codes, drug names, and diagnostic imaging reports to predict surgery. For each prediction task, we evaluated the model’s performance using classical and generalizability evaluation. For classical evaluation, we split the data into training (80%) and testing (20%). For generalizability evaluation, we split the data based on the healthcare system. We used the area under the curve (AUC) to assess performance for each evaluation. We compared results to a benchmark model (i.e. LASSO logistic regression).

**Results:**

For classical performance, the DL model outperformed the benchmark model for early surgery with an AUC of 0.725 compared to 0.597. For late surgery, the DL model outperformed the benchmark model with an AUC of 0.655 compared to 0.635. For generalizability performance, the DL model outperformed the benchmark model for early surgery. For late surgery, the benchmark model outperformed the DL model.

**Conclusions:**

For early surgery, the DL model was preferred for classical and generalizability evaluation. However, for late surgery, the benchmark and DL model had comparable performance. Depending on the prediction task, the balance of performance may shift between DL and a conventional ML method. As a result, thorough assessment is needed to quantify the value of DL, a relatively computationally expensive, time-consuming and less interpretable method.

**Supplementary Information:**

The online version contains supplementary material available at 10.1186/s12911-022-02096-x.

## Introduction

Low back pain (LBP) is one of the most common reasons for a hospital visit, with an annual prevalence of 7.4% [1]. As a result, LBP incurs an annual cost of $100 billion and is the leading contributor to disability and workdays lost [2–4]. Despite numerous available interventions for LBP, it remains difficult to diagnose and treat effectively, in part because LBP has many anatomic and clinical subtypes [5, 6]. Lumbar disc herniation (LDH) and lumbar spinal stenosis (LSS) are two specific spine-related clinical syndromes that are highly associated with LBP [2, 7, 8]. Patients with LDH experience pain caused by extension of the intervertebral disc material beyond the disc space, which may compress adjacent spinal nerves [7, 9]. Patients with LSS experience pain associated with narrowing of the spaces within the spine due to changes in the intervertebral discs and facet joints, which may also compress the spinal nerves [10, 11]. These syndromes have overlap as (1) patients with one entity can develop the other and (2) both involve neuropathic lower extremity pain.

Patients with LDH/LSS are often started with non-surgical treatments and if those are not effective then go on to have decompression surgery to relieve the compressed spinal nerves [11–13]. However, decompression has both potential benefits and risks. Recent studies indicate a possible improvement in early health outcomes due to decompression [14–17], but randomized controlled trials (RCTs) indicate that benefits may decrease over time [14, 15]. Another study found that LDH patients who underwent surgery had better short-term improvement in function and pain relief compared to non-surgical treatments [17]. A RCT found that LSS patients who received decompression surgery instead of non-surgical treatments had better initial improvement in back pain, but this benefit diminished over time [16]. On the other hand, decompression surgery has potential risks, with 18% of LSS patients experiencing adverse events [18], and between 3.1% and 9% having clinical worsening within 1 year [19]. Continuation of non-surgical treatment is the default treatment option for patients with LDH/LSS, as many will improve over time without surgery [20]. Therefore, patients with LDH/LSS may be observed for long periods of time before surgery is considered. In summary, recommendation of decompression surgery is complicated as the outcome can be positive or negative depending on the patient. Early identification of patients at high risk of eventual surgical decompression (i.e. failure of non-surgical treatments) could inform discussions between patients and their clinicians on the benefits and risks of pursuing surgery.

Machine learning (ML) is a promising method to assist patients and healthcare providers to understand a patient’s predicted risk of eventual decompression surgery [21–23]. ML can be used to develop predictive models from large data sets [24, 25]. In recent years, deep learning (DL) has emerged as a popular method to learn low-dimensional representations of raw input data with the potential to improve predictive modeling performance [26]. Several works have applied DL to predict clinical outcomes. Norgeot et al. developed a DL model to predict rheumatoid arthritis [27]. Choi et al. used a recurrent neural network to predict heart failure [28]. These and other similar approaches used structured electronic health record (EHR) data (e.g. diagnosis codes), but with the growing volume and complexity of EHR data, combining structured and unstructured data (e.g. narrative text notes) is gaining acceptance [29]. As a result, multimodal deep learning (MDL—referring to the use of more than one mode of data) has emerged as a possible way to holistically model a patient’s full characteristics [30–32]. However, the performance advantages often observed with deep learning models come with increased computational costs for training and inference relative to traditional machine learning approaches, as well as loss of model interpretability. A recent study indicated that depending on the underlying relationship of the features and outcome, conventional ML methods may provide simpler, cheaper, and more useful data modeling that can achieve comparable, if not better, performance than DL-based methods [33]. Rigorously testing any MDL approach against a conventional ML method is needed to determine whether the additional costs it incurs are truly justified.

**Fig. 1:**
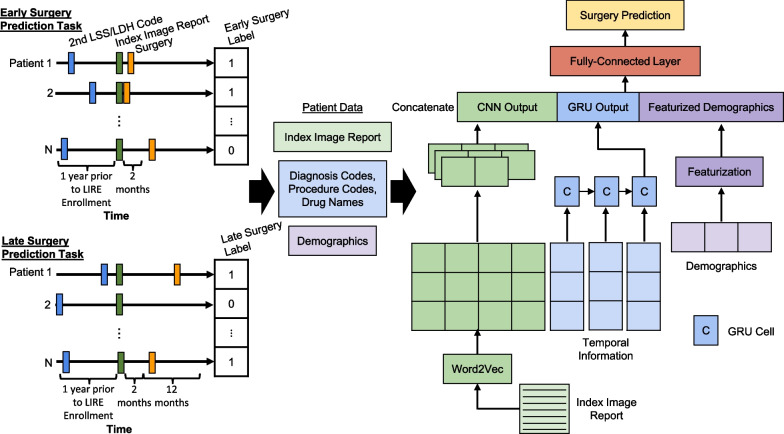
Overview of the prediction pipeline. For early surgery, we identified LDH/LSS patients if they have at least 2 diagnosis codes one year prior to LIRE enrollment and then identified out of these patients as having surgery if they had at least 1 decompression code within 2 months ahead. For late surgery, we identified LDH/LSS patients if they have at least 2 diagnosis codes one year prior to LIRE enrollment and then identified out of these patients as having surgery if they had at least 1 decompression code within 12 months ahead of a 2 month gap. For each prediction task, we collected patients’ demographics, diagnosis codes, procedure codes, drug names, and index image reports. For the multimodal deep learning architecture, the index image reports are passed into a CNN, the diagnosis and procedure codes and drug names are passed into a GRU, and the demographics are featurized. The output from each network are concatenated together along with the featurized demographics and then passed into a fully-connected layer and then to an output layer to make predictions. CNN, Convolutional Neural Network; GRU, Gated Recurrent Unit; LSS, Lumbar Spinal Stenosis; LDH, Lumbar Disc Herniation; LIRE, Lumbar Imaging With Reporting Of Epidemiology

In the current study, we aim to predict early (within 2 months) and late (within 12 months after a 2 month gap) decompression surgery for patients with LDH/LSS by applying MDL to their structured and unstructured data and comparing the performance to a benchmark model, LASSO logistic regression (Fig. 1). The ability to identify patients at high risk of ultimately needing surgery accurately could lead clinicians to either try more focused or intensive non-surgical treatments, or recommend surgery earlier than they otherwise would. Additionally, patients predicted as unlikely to receive surgery may be motivated to continue with their non-surgical treatment plan.

## Methods

### Data source

This was a retrospective study that utilized the Lumbar Imaging with Reporting of Epidemiology (LIRE) study dataset which consisted of approximately 250,000 patients from four healthcare systems (Group Health, Kaiser Permanente Northern California, Henry Ford, and Mayo Clinic) who received a thoracic or lumbar spine plain X-ray, magnetic resonance imaging (MRI), or computed tomography (CT) between October 1, 2013 and September 30, 2016 [34]. The LIRE study was a multicenter intervention study that investigated whether inserting text about the prevalence of common imaging findings into lumbar spine imaging reports reduced subsequent spine-related interventions [34]. Once enrolled in the study, EHR data was collected from patients for two years following and one year prior to their first (i.e. index) imaging.

### Patient selection

From the LIRE dataset, we selected patients who had at least two occurrences of International Classification of Diseases (ICD)-9 or ICD-10 codes related to LSS or LDH (Additional file 1: Table S1). This criterion was agreed upon by our clinical experts (PS, JF, and JGJ), to increase confidence in identifying patients with these syndromes [35, 36]. We based our ICD codes on two previous studies [37, 38]. Martin et al. selected ICD-9 codes that were commonly used to describe spine-related problems. These codes were identified by searching the annual updates published by the World Health Organization and referencing the Conversion Tables of new ICD-9 codes published by the National Center for Health Statistics to help identify newly added or modified codes [37]. They then validated their process to group patients based on these codes by comparing it to clinician judgment using sensitivity and specificity analysis. Deyo et al. further grouped their patients with back pain into back and leg pain or herniated disc and lumbar stenosis groups based on ICD-9 codes [38]. We updated the code lists of Martin et al. and Deyo et al. to also include ICD-10 [39].

### Outcome

We further split patients with LDH/LSS into two prediction tasks: early and late surgery (Fig. 1). We chose these outcomes based on the clinical rationale that early surgery for LDH/LSS is more likely driven by severe or progressive neurologic deficits, as opposed to late surgery, which is more likely to be driven by chronic pain [9]. For early surgery, we limited the patients to those that had at least two LDH/LSS diagnosis codes within the year prior to LIRE enrollment and then searched two months ahead for the presence (positive) or absence (negative) of their first decompression surgery code. We had 198 (2.4%) LDH/LSS patients in the positive group and 8189 (97.6%) LDH/LSS patients in the negative group. For late surgery, we limited patients to those that had at least two LDH/LSS diagnosis codes within the year prior to LIRE enrollment then searched, after a two month gap, one year ahead for the presence or absence of their first decompression surgery code. We had 431 (5.0%) LDH/LSS patients in the positive group and 8189 (95.0%) LDH/LSS patients in the negative group. There was no overlap of patients with early and late decompression surgery. The decompression phenotype was developed by manually reviewing lists of Current Procedural Terminology (CPT) and ICD-9 Procedure Coding System that were potentially associated with surgery by at least one non-clinician reviewer (Additional file 1: Table S1) [34, 40, 41]. Any uncertain codes were also reviewed by two clinician reviewers (PS and JF) and discussed until consensus was achieved by both reviewers.

### Features

We considered patient demographics, diagnoses, procedures, prescription information, and radiology reports as predictors for the model (Fig. 1). For demographics, we considered patients’ race, age, healthcare system, and ethnicity. For the primary care provider for each patient, we considered their gender, type of clinician, and speciality. For diagnosis, we considered patients’ ICD-9 and ICD-10 codes and the day they received the diagnosis. For procedures, we considered patients’ CPT and Healthcare Common Procedure Coding System Level II codes (i.e. procedure codes) and the day they received their procedure code. For prescriptions, we considered the drug name and prescription day. For radiology reports, we considered the finding and impression sections from the index imaging report in the LIRE study along with the type of image (i.e. X-ray, CT, or MRI).

### Preprocessing/featurization

#### Demographics

**Fig. 2:**
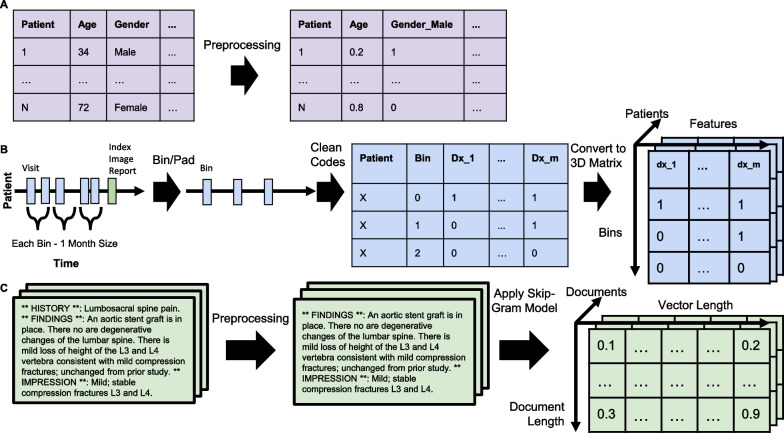
Visualization of data preprocessing for deep learning. **A** For the demographics data (i.e. static data), we created dummy variables for the categorical features and normalized the discrete numerical feature (i.e. age) at the patient level. **B** For the diagnosis, procedures, and drug names data for the deep learning model (i.e. temporal data), we limited the information to the last three months of information prior to the index image for both prediction tasks. We cleaned up the ICD codes by mapping them to level three in the hierarchy. To maintain the same number of bins (i.e. three), we added empty bins to patients with less than three bins. Finally, we converted the dataframe into a 3D tensor. **C** We pre-trained a skip-gram model on 123,461 LIRE reports. We applied our model to each index imaging report to extract a feature representation. ICD, International Classification of Diseases; LIRE, Lumbar Imaging with Reporting of Epidemiology

This information is composed of patient and provider demographics along with the type of index image. To convert the data into a format for ML, we created dummy variables for the categorical features and normalized the discrete numerical feature (i.e. age) at the patient level (Fig. 2A). For early surgery, there are 23 features, while for late surgery there are 22 features.

#### Diagnosis, procedures, and prescriptions

We limited temporal data (diagnosis, prescriptions, and procedures) to the last three months of information prior to the index image for both prediction tasks, so that across the patients we (1) ensure that the time period is consistent and (2) minimize the variability in the amount of available data. The purpose was to minimize any influence from the heterogeneity of these factors on the prediction tasks. For diagnosis codes, we mapped ICD-10 to equivalent ICD-9 codes to minimize redundancy and then assigned all ICD-9 codes to depth level three on the ICD hierarchy using crosswalk files from cms.gov. We chose depth level three (i.e. the first three digits of ICD codes) to reduce the feature space, but also maintain an informative level of granularity [42]. ICD codes are organized into a hierarchy based on shared clinical characteristics. The further down in this hierarchy, the more specific the disease based on anatomic site, etiology, and manifestations.

##### Featurization for classical machine learning

We created dummy variables for the features (i.e. diagnosis codes, procedure codes, and drug names) at the patient-level. Further, we excluded extremely rare (≤ 0.1%) or common (≥ 99%) features to reduce the feature space. For early surgery, there are 25 features for diagnosis, 103 features for prescriptions, and 71 features for procedures. For late surgery, there are 25 features for diagnosis, 106 features for prescriptions, and 72 for procedures.

##### Featurization for deep learning

We binned the data into one month intervals to reduce the sparsity of the eventual temporal feature matrix. We then created dummy variables for the features (i.e. diagnosis codes, procedure codes, and drug names) at the bin-level for each patient. To maintain the same number of bins (i.e. three), we added empty bins to patients with less than three bins. Finally, we converted the dataframe into a 3D tensor where the depth corresponds to the number of the patients, the height to the number of bins, and the width to the number of unique features (Fig. 2B). For early surgery, there are 41 features for diagnosis, 245 features for prescriptions, and 160 for procedures. For late surgery, there are 43 features for diagnosis, 245 features for prescriptions, and 161 features for procedures.

#### Index imaging reports

We developed regular expressions to search for the headers of the finding and impression sections by reviewing a subset of these reports. For all our reports, we applied our regular expressions then isolated and concatenated the accompanying text in these sections. The purpose was to limit the text to only information that pertained to the diagnostic image itself. We then cleaned the text by converting it to lowercase, removing punctuation, removing extra whitespace, removing stopwords, and then isolated the stem of each word using a PorterStemmer from the python package nltk [43].

##### Featurization for classical machine learning

We extracted uni-, bi-, and trigrams from the cleaned text using the python package scikit-learn [44]. Further, we excluded extremely rare (≤ 0.1%) or common (≥ 99%) n-grams to reduce the feature space. For early surgery, there are 26,245 features, while for late surgery there are 26,983 features.

##### Featurization for deep learning

To convert the index reports into a format for the DL architecture, we used the python package genism [45]. We first collected reports (n = 123,461) post LIRE enrollment and preprocessed them the same way as the index reports. We pre-trained a skip-gram model with a vector length set to 300 on these reports. Parameter values and architecture were based on a recent study that evaluated different types of word2vec architectures and observed that this architecture and values lead to optimal performance when converting radiology reports into embedding representations [46, 47]. We extracted the vocabulary and the associated embeddings from this pre-trained skip-gram model (Fig. 2C). To maintain the same length for each document (a requirement for efficient batch-based deep learning implementations), we padded reports to the maximum length across index reports: 559 for early surgery and 573 for late surgery. We chose this approach to ensure the impression section was included as it summarizes the key findings from the image [48].

### Machine learning

#### Benchmark model

We used the LASSO [49] logistic regression built using the python package scikit-learn and weighted the positive and negative group inversely proportional to their prevalence to address the imbalance in our dataset. Because the data naturally has multicollinearity among different features (i.e. diagnosis codes, procedure codes, and prescriptions), this can lead to over- and underestimating relationships between the features and outcome. As a result, we chose LASSO since it performs feature selection through penalization to minimize these redundant features. To identify the optimal regularization parameter (lambda), we performed fivefold cross validation within the training set. We chose the lambda value that led to the highest average F1-score across the folds to shrink the coefficients of the features. We chose the F1-score since it’s a popular performance metric for imbalanced datasets, which takes into consideration how well the model can capture the positive group (i.e. minority group), but also the reliability of these positive predictions. Because LASSO’s lambda value and its subsequent performance can be affected by how the data is split, we repeated the process of fivefold cross validation 50 times, each process with a different split of the data into the folds, then chose the prevalent lambda value across repeats [50]. Additionally, to assess the value of each modality, we repeated this process for each data type by itself (i.e. codes, demographics, and textual).

#### Multimodal deep learning model

The MDL architecture was built using the python package PyTorch and is composed of three entities: 1-layer Convolutional Neural Network (CNN), 1-layer Gated Recurrent Unit (GRU), and two 1-layer Fully-Connected (FC) (Fig. 1) [51]. This architecture is based on the work by Zhang et al., which compared two different MDL architectures that differed in the use of either a CNN or Long Short-Term Memory (LSTM) for both sequences of clinical notes and structured data [30]. Since in our approach we do not have sequences of clinical notes, this comparison is out of scope. Additionally, we decided to use a GRU instead of an LSTM since the former is a simpler architecture, but can lead to similar performance [52, 53]. We passed the featurized index reports and the pre-trained skip-gram embeddings and vocabulary into a CNN, the featurized temporal data into a GRU, concatenated the output from these individual networks with the featurized demographics and then passed the resulting concatenated vector to the FC layer to make predictions. We included a FC layer to convert the temporal input into embeddings before passing into the GRU as previous studies of this approach showed improvement in prediction performance [54–56]. We used a CNN, because we wanted to model the spatial relationship of the words in our reports in relation to our prediction task. The MDL model was trained using the Adam optimizer with a weight decay and ReLU as the activation function. We used Cross Entropy Loss as the loss function with weighting of the positive and negative group inversely proportional to their prevalence to address the imbalance in our dataset [57]. We minimized subsets of weights from co-adapting (i.e. overfitting to the noise in the training data) by adding a dropout to the hidden layer of the FC to allow all weights to participate in the prediction task [58]. To optimize the hyperparameters (i.e. number of filters, learning rate, dropout rate, GRU hidden size, and weight decay), we 1) split the training data into 80% for training and 20% for validation, 2) used previous works as a starting point for values [30, 59], then 3) grid searched to identify the combination of values that was associated to the lowest validation loss (Additional file 2: Table S2). We trained our model for 30 epochs using a learning rate scheduler to decrease the learning rate value when the validation loss increased to avoid overfitting. During the training process, our model was allowed to fine-tune the pre-trained skip-gram embedding values. Unlike the LASSO optimization, we did not perform fivefold cross validation as it would have been prohibitively computationally expensive. Additionally, we repeated this entire process for each individual network (i.e. 1-layer FC, 1-layer GRU with 1-layer FC, and 1-layer CNN with 1-layer FC) in the MDL architecture by itself and its associated data: demographics, temporal, and textual, respectively.

### Evaluation

#### Classical

For each prediction task’s dataset, we split it into a training (80%) and test set (20%). After hyperparameter tuning, the LASSO models were retrained on the full training set using optimized lambda values, while the DL models were retrained on the same training and validation set using the optimized hyperparameter values. The reason for this is that the learning rate scheduler for the DL models needs to monitor the validation loss, so that it can properly update the training process. We then evaluated the models’ performance on the test set using the performance metrics: recall, specificity, balanced accuracy, precision, F1-score, area under the curve (AUC), and area under the precision-recall curve (AUPRC). While we calculated these different performance metrics, we prioritized AUC in the analysis and interpretation since it’s (1) a global metric that assesses overall performance across different thresholds and (2) a more popular metric in the biomedical ML field. We estimated the significance of differences in performance between models by performing a t-test on 1000 bootstrapped test samples [27, 31]. We used a Bonferroni correction to correct for multiple hypothesis testing when comparing MDL to the three individual networks by multiplying each *p* value by three.

#### Generalizability

For generalizability, we divided the data based on the healthcare system. We trained the models on Kaiser Permanente Northern California and tested on the remaining systems. We chose Kaiser Permanente Northern California as the training set, since it made up roughly 80% of our entire dataset. For the test set, we excluded the Mayo Clinic since it contained a substantially smaller number of patients compared to Henry Ford and Group Health (Table 2). For each test system, we then evaluated the models’ performance using the performance metrics: recall, specificity, balanced accuracy, precision, F1-score, AUC, and AUPRC. As before, while we calculated these different performance metrics, we prioritized AUC when interpreting results. We estimated the significance of performance differences between models by bootstrapping 1000 samples for each healthcare system in the test set and then calculating the performance metrics. For each metric and »healthcare system, we performed a t-test comparing the distributions between the models. We used a Bonferroni correction to correct for multiple hypothesis testing when comparing MDL to the three individual networks by multiplying each *p* value by three.

## Results

### Data characteristics

For early surgery, we identified 8387 patients with a prevalence of 2.4% for decompression surgery (Table 1). For late surgery, we identified 8620 patients with a prevalence of 5.0% for decompression surgery. For the early surgery dataset, the average age was 57 years, while for late surgery it was 57.2 years. Both datasets were balanced for gender with females representing 56.2%. The majority of patients from both datasets were (1) white, 63.4% and 63.8%, respectively; and (2) from Kaiser Permanente Northern California, 84.3% and 84.4%, respectively. We found that the majority of patients in both datasets had an MRI with prevalence of 69.3% and 69.4%, respectively.

**Table 1:** Data characteristics

Characteristics	Early surgery	Late surgery
N	8387	8620
Negative	8189 (97.6%)	8189 (95.0%)
Positive	198 (2.4%)	431 (5.0%)
Average days between LIRE enrollment and decompression surgery	34.3	168
Age	57	57.2
Gender		
Female	4713 (56.2%)	4845 (56.2%)
Race		
White	5317 (63.4%)	5502 (63.8%)
Black	991 (11.8%)	1007 (11.7%)
Unknown	990 (11.8%)	1000 (11.6%)
Asian	928 (11.1%)	948 (11.0%)
Pacific Islander	50 (0.6%)	51 (0.6%)
Other	27 (0.3%)	26 (0.3%)
Multiracial	17 (0.2%)	19 (0.2%)
Ethnicity		
Not available	5945 (70.9%)	6129 (71.1%)
Not Hispanic	1233 (14.7%)	1263 (14.7%)
Hispanic	1209 (14.4%)	1228 (14.2%)
Image type		
MRI	5810 (69.3%)	5980 (69.4%)
X-ray	2517 (30.0%)	2576 (29.9%)
CT	60 (0.7%)	64 (0.7%)
System		
Kaiser Permanente	7071 (84.3%)	7274 (84.4%)
Henry Ford	654 (7.8%)	657 (7.6%)
Group Health	486 (5.8%)	517 (6.0%)
Mayo Clinic	176 (2.1%)	172 (2.0%)

### Classical performance assessment

**Fig. 3:**
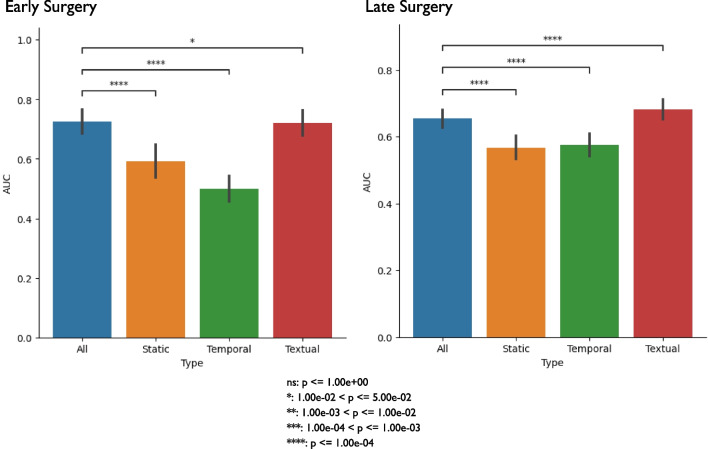
Classical performance assessment of multimodal deep learning against individual networks. We compared the performance of the MDL architecture against each individual network (i.e. temporal, textual, and demographics). We calculated 1000 bootstrap samples from the test set. For each sample, we calculated AUC. Finally, for each prediction task, we performed a t-test to assess significance between the model that contained all three data types and the models using a single data type; we indicate significance with an asterisk. We corrected for multiple hypothesis testing using Bonferroni correction by multiplying each *p* value by three. AUC, Area Under the Curve; MDL, Multimodal Deep Learning

To assess the best performing model for each prediction task, we trained and tested each model, then calculated performance metrics on the test set, and then used a t-test to assess significant performance differences. For early surgery, we found that MDL had a significantly higher AUC (0.725) compared to the benchmark model (0.597) (Table 2). For late surgery, we found that MDL had a significantly higher AUC (0.655) than the benchmark’s AUC of 0.635 (Table 2). For both early and late surgery, we found that textual data (i.e. index image reports) was the main contributing factor to MDL’s performance based on comparing performances (Fig. 3).

**Table 2:** Classical performance assessment of multimodal deep learning against benchmark

Prediction	Prevalence	N	Model	Recall	Precision	Balanced accuracy	F1	AUC	AUPRC
Early Surgery	0.024	824	MDL	0.300 ± 0.077*	0.086 ± 0.021*	0.610 ± 0.039*	0.133 ± 0.033*	0.725 ± 0.040*	0.061 ± 0.014*
			Benchmark	0.375 ± 0.076	0.069 ± 0.014	0.624 ± 0.038	0.116 ± 0.023	0.597 ± 0.050	0.047 ± 0.011
Late Surgery	0.049	851	MDL	0.595 ± 0.051*	0.080 ± 0.007*	0.619 ± 0.026*	0.140 ± 0.012*	0.655 ± 0.026*	0.077 ± 0.009*
			Benchmark	0.440 ± 0.056	0.076 ± 0.009	0.580 ± 0.028	0.129 ± 0.016	0.635 ± 0.031	0.079 ± 0.011

We compared the performance of the MDL architecture against the benchmark (i.e. LASSO). We calculated 1000 bootstrap samples from the test set. For each sample, we calculated the performance metrics: recall, specificity, balanced accuracy, precision, F1-score, AUC, and AUPRC. We then calculated the average and standard deviation across the samples. For each prediction task, we underline the model that had the best performance for each metric. Finally, we performed a t-test to assess significance between each model’s performance metrics for each prediction task; we indicate significance with an asterisk

AUC, Area Under the Curve; AUPRC, Area Under the Precision-Recall Curve; MDL, Multimodal Deep Learning

### Generalizability performance assessment

**Fig. 4:**
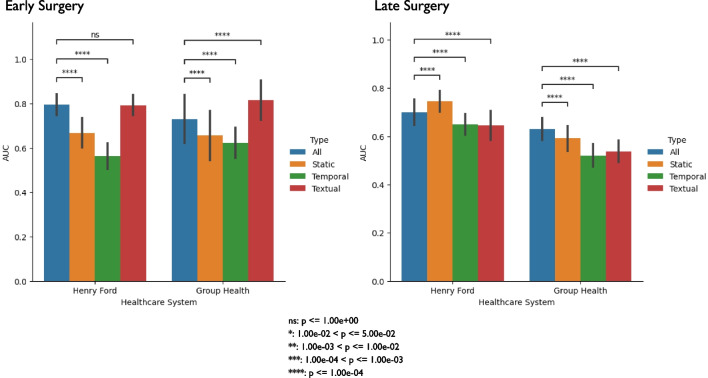
Generalizability performance assessment of multimodal deep learning against individual networks. We compared the performance of the MDL architecture against each individual network (i.e. temporal, textual, and demographics) for each system. We calculated 1000 bootstrap samples from the test set. For each sample, we calculated AUC. Finally, for each prediction task and system, we performed a t-test to assess significance between the model that contained all three data types and the models using a single data type; we indicate significance with an asterisk. We corrected for multiple hypothesis testing using Bonferroni correction by multiplying each *p* value by three. AUC, Area Under the Curve; MDL, Multimodal Deep Learning

To assess the most generalizable model for each prediction task, we trained on Kaiser Permanente Northern California data and tested on the remaining healthcare systems. We excluded Mayo Clinic from the test set since it contained a substantially smaller set of patients compared to Group Health and Henry Ford (Table 1). For early surgery, we found MDL had a significantly higher AUC compared to the benchmark model for both healthcare systems, 0.731 compared to 0.656 for Group Health and 0.795 compared to 0.714 for Henry Ford (Table 3). For late surgery, we found that the benchmark had a significantly higher AUC compared to MDL for both healthcare systems, 0.641 compared to 0.630 for Group Health and 0.707 compared to 0.700 for Henry Ford (Table 3). Similar to classical performance, we found that textual data mainly contributed to MDL’s generalizability performance for early surgery, but for late surgery, all three data types seemed to contribute, with a marginal advantage for static data, to MDL’s generalizability performance (Fig. 4).

**Table 3:** Generalizability performance assessment of multimodal deep learning against benchmark

Prediction	System	Prevalence	N	Model	Recall	Precision	Balanced Accuracy	F1	AUC	AUPRC
Early Surgery	Group Health	0.021	239	MDL	0.600 ± 0.161*	0.075 ± 0.020*	0.720 ± 0.081*	0.132 ± 0.036*	0.731 ± 0.109*	0.105 ± 0.050*
				Benchmark	0.300 ± 0.152	0.056 ± 0.028	0.595 ± 0.076	0.094 ± 0.047	0.656 ± 0.113	0.149 ± 0.114
	Henry Ford	0.039	324	MDL	0.640 ± 0.097*	0.127 ± 0.021*	0.732 ± 0.050*	0.212 ± 0.033*	0.795 ± 0.047*	0.128 ± 0.031*
				Benchmark	0.200 ± 0.079	0.087 ± 0.034	0.557 ± 0.040	0.120 ± 0.047	0.714 ± 0.050	0.088 ± 0.023
Late Surgery	Group Health	0.079	254	MDL	0.425 ± 0.079*	0.143 ± 0.026*	0.603 ± 0.041*	0.214 ± 0.038*	0.630 ± 0.046*	0.120 ± 0.020
				Benchmark	0.600 ± 0.080	0.109 ± 0.014	0.590 ± 0.042	0.185 ± 0.024	0.641 ± 0.044	0.119 ± 0.023
	Henry Ford	0.042	325	MDL	0.482 ± 0.099*	0.085 ± 0.017*	0.628 ± 0.051*	0.145 ± 0.029*	0.700 ± 0.053*	0.091 ± 0.024*
				Benchmark	0.556 ± 0.096	0.112 ± 0.019	0.682 ± 0.048	0.186 ± 0.031	0.707 ± 0.057	0.097 ± 0.022

We compared the generalizability performance of the MDL architecture and the benchmark (i.e. LASSO). For each test system, we evaluated models’ performance using the performance metrics. We estimated significance performance between models by bootstrapping 1000 samples for each test system. For each prediction task and system, we performed a t-test comparing the bootstrapped samples between the two models across the performance metrics; we indicate significance with an asterisk for the MDL row. We underline the model that had the best average performance for each metric for each system

AUC, Area Under the Curve; AUPRC, Area Under the Precision-Recall Curve; MDL, Multimodal Deep Learning

## Discussion

Early identification of LDH/LSS patients at high risk of eventual surgical decompression (i.e. failure of non-surgical treatments) could inform discussions between healthcare providers and patients on the benefits and risks of pursuing surgery using information specific to each patient. In our study, we developed a MDL model that leveraged textual, temporal, and demographic information to predict decompression surgery for LDH/LSS patients and then evaluated classical and generalizability performance against a benchmark model. For early surgery, MDL was the better performing model for both evaluations. For late surgery, MDL was the better performing model for classical performance, however for generalizability the benchmark model was better performing. While the difference in performance between MDL and the benchmark model for predicting late surgery was statistically significant, it was not necessarily meaningful due to the small magnitude. This stands in contrast to the larger differences in performance observed when MDL had the advantage in early surgery. Our study suggests that in some tasks, MDL and the benchmark conventional ML method can have similar performance, while in others (i.e. early surgery) MDL has a clear advantage. As a result, thorough assessment is needed to quantify the value of DL, a computationally expensive and time-consuming method that is relatively difficult to interpret.

For classical performance evaluation, the MDL models achieved a mean AUC of 0.725 for early surgery and 0.655 for late surgery. The early surgery performance approaches results from prior studies that used DL to predict aspects of lumbar surgeries [60, 61]. André et al. assessed the feasibility of training a DL model on synthetic patients generated from EHR data to predict the positive and negative outcomes from decompression surgery resulting in an AUC of 0.78, while Wilson et al. predicted spinal surgery by applying deep learning to MRI images and achieved an AUC of 0.88. The difference in our results can be attributed to (1) our larger dataset (2) our different outcomes, (3) Andre et al. using synthetic patients, rather than real patients, and (4) Wilson et al. used only imaging data. As a result, these studies’ results are limited in their generalizability, and their results are not strictly comparable to ours. Nonetheless, they provide some context for interpretation of the performance of our models. Of note, a previous study by Keeney et al. used logistic regression to predict which Washington State workers with disability claims for back injuries would receive lumbar spine surgery (i.e. decompression, fusion, and/or both) or not, with an AUC of 0.93 [62]. This AUC value exceeds that from our benchmark and DL models for both early and late surgery. Keeney et al. found that the driving feature for this performance was a binary feature indicating whether a patient’s injury was first seen by a surgeon or not, and speculated that this may indicate that “who you see is what you get” [62]. This suggests that information about providers (which was not available in our dataset) may have further improved our models’ performance. However, the inclusion of provider type validates what is already known [63, 64], while our approach further explored possible new associations by being more holistic about patients’ data.

Our study fills an important gap in the literature by evaluating the generalizability of a predictive model for spine surgery, a domain in which such evaluations are rare [65]. As noted, MDL was the most generalizable model for both prediction tasks, with implications for the development of models for broad deployment. Our rigorous evaluation shows DL-based models can learn a generalizable representation from the training data that can be applied to other healthcare systems’ datasets. As noted in Azad et al., if we want to bring ML models into the clinical space, more external validation is needed to prove that performance is not specific to the internal datasets used for training and testing [65].

Textual data (i.e. the index image report) was the contributing data type for the MDL model for early and late surgery. This same observation was seen in the benchmark models’ top and bottom 10 predictors as well (Additional file 3: Tables S3 and Additional file 4: Table S4). As noted earlier, early surgery for LDH/LSS is more likely driven by severe or progressive neurologic deficits, as opposed to late surgery, which is more likely to be driven by chronic pain. The drivers for both surgeries seem to be anatomic findings that may be associated with a greater likelihood of pain or persistent pain. While neurologic deficits cannot be known from textual radiology report data, the anatomic findings were captured. It is possible that our diagnosis codes could not fully represent these neurologic deficits and our models could have performed better if clinical notes (which may mention neurologic deficits) were included as another data source for ML, however this information was not captured in the LIRE study. Additionally, for late surgery, we observed that textual alone had a significantly higher AUC compared to using all the data types for DL (Fig. 3). This observation is most likely due to the fact that for a given prediction task in ML, more features does not necessarily mean better performance as the distinction between positive and negative labels can get difficult to discern with noisy features (i.e. non-textual data) vs. using a smaller set of useful features (i.e. textual data) as seen in other ML studies [66].

Our study highlights the potential disparities in spine care. For early surgery, static-only DL had a higher AUC than temporal-only DL (Fig. 3). This same observation was seen in our benchmark models; the static-only model was mainly driven by sociodemographic factors such as age, sex, race, ethnicity, and healthcare system, while the temporal-only model reflected clinical characteristics (Additional file 5: Table S5). This is consistent with other work related to back pain and spine surgery, where sociodemographics provide considerable predictive information, and our group has previously shown that age, sex, race, and ethnicity are all associated with health care utilization in back pain and spinal conditions [67, 68]. Even for our late surgery benchmark model that utilized all data types, “White” was a top feature (Additional file 4: Table S4). These findings underscore concerns about disparities in spine care associated with race and ethnicity [69]. Additionally, the temporal features included in these EHR-based analyses may not be able to capture important time-varying clinical factors such as increases in pain intensity and/or evolving neurologic deficits, which are expected to confer an increased risk of surgical decompression.

There are several limitations to this study. First, expanding our hyperparameter value search space could have improved our DL-based models’ performances, however we used prior studies to focus our grid search on the most important hyperparameters and their ranges of values on account of constraints on computational resources. Second, we only used DL and logistic regression for our ML models and did not consider other methods. Including more conventional ML methods might have provided better performance than logistic regression and even DL. However, our objective was to specifically use DL to predict surgery and benchmark this costly method against the most popular and accessible method for researchers: logistic regression. Third, a bias in medicine is that sicker patients generally have more data points than healthier patients. We sought to address this by limiting the patients’ data to the last three months and then binned into one month intervals, so that across the patients we 1) ensure that the time period is consistent and 2) minimize the variability in the amount of available data.

## Conclusions

In summary, we built a MDL architecture to predict early and late decompression surgery for LDH/LSS patients. For each prediction task, we compared this architecture’s performance within and across different healthcare systems against LASSO logistic regression, a conventional ML method. Our rigorous testing shows that depending on the prediction task, DL can significantly outperform a conventional ML method or both have comparable performances. This shows that thorough assessment is needed to validate the need for DL over using a conventional ML method. Finally, based on our MDL model’s high AUC and low AUPRC, it can be used as a decision support tool to assist clinicians by mediating early discussions with their patients about possible treatments.

## Supplementary Information

**Additional file 1: Table S1.** List of Codes for Lumbar Stenosis, Lumbar Disc Herniation, and Decompression

**Additional file 2: Table S2.** Hyperparameter Search Space

**Additional file 3: Table S3.** Top 10 and Bottom 10 Predictors for Early Surgery Benchmark Model for Classical Performance

**Additional file 4: Table S4.** Top 10 and Bottom 10 Predictors for Late Surgery Benchmark Model for Classical Performance

**Additional file 5: Table S5.** All Ordered Features with Non-Zero Coefficient for Static- and Temporal-Only Early Surgery Benchmark Models for Classical Performance.

## Data Availability

The de-identified dataset analyzed during the current study is available through a private archive hosted by the Resource Core of the University of Washington Clinical Learning, Evidence And Research (CLEAR) Center for Musculoskeletal Disorders upon request (https://theclearcenter.org/about/resource-core/). The request will be reviewed by the CLEAR Center Resource Core Director and Associate Director for scientific soundness. Representatives of the LIRE data collection sites will also have the opportunity to review and approve requests. Costs of proposal review and data preparation will be borne by the requester. For further questions about the request, please contact Jeffrey G. Jarvik, jarvikj@uw.edu.

## Abbreviations

LBP: Lower Back Pain
LDH: Lumbar Disc Herniation
LSS: Lumbar Spinal Stenosis
RCT: Randomized Controlled Trial
ML: Machine Learning
DL: Deep Learning
EHR: Electronic Health Record
MDL: Multimodal Deep Learning
LIRE: Lumbar Imaging with Reporting of Epidemiology
MRI: Magnetic Resonance Imaging
CT: Computed Tomography
ICD: International Classification of Diseases
CPT: Current Procedural Terminology
CNN: Convolutional Neural Network
GRU: Gated Recurrent Unit
FC: Fully-Connected
LSTM: Long Short-Term Memory
AUC: Area Under the Curve
AUPRC: Area Under the Precision-Recall Curve

## Code availability

https://github.com/chethanjjj/LowerBackPain-Surgery-Prediction.
